# Energy sources in percutaneous surgery. Are lasers ready to become the gold standard? A narrative review

**DOI:** 10.1590/S1677-5538.IBJU.2026.0196

**Published:** 2026-06-30

**Authors:** João Pedro Leal Puppin, Vinicius Ravena Lourenço, Anderson Pellanda, Alexandre Danilovic, Fabio Carvalho Vicentini, Carlos Alfredo Batagello, Fabio C.M. Torricelli, Rodrigo Perrella, Giovanni S. Marchini, William C. Nahas, Eduardo Mazzucchi

**Affiliations:** 1 Faculdade de Medicina da Universidade de São Paulo Hospital das Clínicas São Paulo SP Brasil Hospital das Clínicas. Faculdade de Medicina da Universidade de São Paulo – HCFMUSP, São Paulo, SP. Brasil; 2 Hospital Militar de Área de São Paulo São Paulo SP Brasil Hospital Militar de Área de São Paulo, São Paulo, SP, Brasil; 3 Instituto do Câncer do Estado de São Paulo São Paulo SP Brasil Instituto do Câncer do Estado de São Paulo – ICESP, São Paulo, SP, Brasil

**Keywords:** Nephrolithotomy, Percutaneous, Review Literature as Topic, Minimally Invasive Surgical Procedures

## Abstract

**Introduction:**

Nephrolithiasis is a prevalent and recurrent condition affecting approximately 10–11% of the population. Percutaneous nephrolithotomy (PCNL) and mini-PCNL are established treatments for large renal stones. Outcomes are influenced by the choice of lithotripsy energy source. Available technologies include pneumatic, ultrasonic, Holmium:YAG (Ho:YAG), and thulium fiber laser (TFL), each with specific advantages and limitations. This review summarizes current evidence on these energy sources in PCNL and mini-PCNL.

**Materials and methods:**

A narrative review was performed using PubMed and Cochrane Library, including studies published between 2014 and 2024. The search included terms related to PCNL and lithotripsy energy sources. Studies involving pediatric populations, anomalous kidneys, or non-standard energy sources were excluded. After screening 95 articles, 32 studies were included, of which 13 provided comparative data on different energy sources and were analyzed.

**Results:**

Differences were observed in operative time, stone-free rates (SFR), bleeding, transfusion rates, complications, and hospital stay. In PCNL, ultrasonic and Ho:YAG lithotripsy showed comparable outcomes, while pneumatic demonstrated slightly lower SFR. In mini-PCNL, ultrasonic and laser technologies yielded similar efficacy. TFL displayed shorter operative time and reduced bleeding and transfusion rates compared to Ho:YAG, although many differences did not reach statistical significance.

**Conclusion:**

Ultrasonic and Holmium:YAG lithotripsy provide comparable outcomes in PCNL, while pneumatic devices may correlate with slightly lower stone-free rates. In mini-PCNL, ultrasonic and laser modalities show similar outcomes. TFL demonstrates potential advantages in operative time and bleeding, however, evidence remains heterogeneous, and further high-quality studies are needed.

## INTRODUCTION

Nephrolithiasis is a common condition affecting millions of individuals worldwide. Epidemiological data from the United States demonstrate a marked increase in both incidence and prevalence over recent decades. Analyses from the National Health and Nutrition Examination Survey (NHANES) estimate a kidney stone prevalence of approximately 10–11%, with an annual incidence approaching 1%. Although rates remain higher in men (1), the incidence among women has increased progressively, suggesting a narrowing gender gap (2). Historically, prevalence increased from 3.8% in the late 1970s to 5.2% in the early 1990s, highlighting a sustained upward trend over time (3). Globally, urolithiasis affects approximately 116 million individuals currently, particularly in low- and middle-income countries, reflecting the impact of urbanization, dietary changes, and metabolic risk factors (4). Furthermore, kidney stone disease is characterized by a high recurrence rate, with approximately 30–50% of patients experiencing recurrence within 5 years (5), reinforcing its chronic and relapsing nature.

Percutaneous nephrolithotomy (PCNL) is the standard treatment for large renal stones (6). Since its introduction in the 1970s, the technique has undergone notable evolution, particularly with the development of miniaturized approaches such as mini-percutaneous nephrolithotomy (mini-PCNL). These techniques aim to reduce surgical morbidity while maintaining efficiency. Compared with standard PCNL, mini-PCNL is associated with shorter hospital stay and lower bleeding rates, although it may be associated with longer operative times (7) (Figure-1).

**Figure 1:**
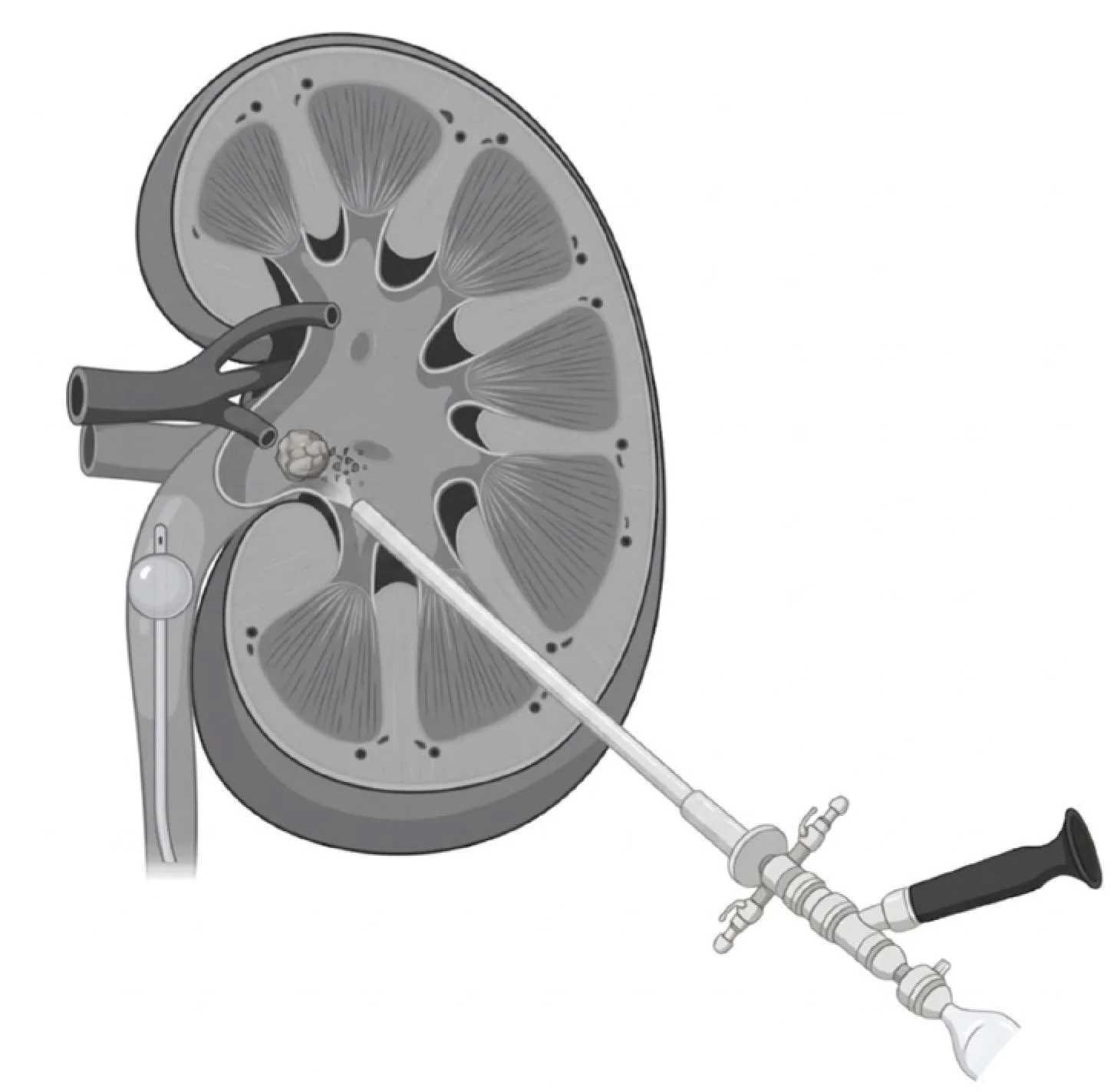
Schematic illustration of laser lithotripsy during percutaneous nephrolithotomy (PCNL).

One crucial factor for the success of PCNL and mini-PCNL is the selection of the appropriate energy sources for lithotripsy, as each modality presents specific advantages and limitations. The main energy sources currently used include pneumatic (ballistic), ultrasonic, and laser technologies like Holmium:YAG and Thulium laser (6, 8).

Pneumatic lithotripsy relies on the rapid forward movement of a projectile, generating a jackhammer effect on the stone. This mechanism may be driven by compressed air, carbon dioxide, or electrokinetic energy. It is considered a safe and effective modality, particularly for the fragmentation of harder stones (9, 10).

Ultrasonic lithotripsy is based on the transmission of ultrasonic vibrations generated by a piezoceramic crystal to a metallic probe in direct contact with the stone. This technique is low-cost, highly effective, and associated with minimal tissue injury, with the additional advantage of allowing simultaneous fragmentation and aspiration of stone debris (8). However, its efficacy may be reduced in certain stone compositions, such as cystine and calcium oxalate monohydrate, as well as in smoother calculi (9).

Lasers represent an alternative energy sources for lithotripsy in PCNL, acting through the thermal expansion and vaporization of water within the calculus (photothermal effect). Two main laser systems are currently used for stone treatment: the Holmium:YAG laser and the thulium laser (11, 12).

The Holmium:YAG laser is generated by excitation of holmium ions embedded in a yttrium–aluminum–garnet (YAG) crystal, typically using a flashlamp, resulting in the emission of pulsed infrared laser energy. High-power lasers (>30 Hz) are generally associated with the production of fine particles that can be cleared spontaneously, whereas low-power lasers (≤15–20 Hz) tend to generate larger fragments requiring active retrieval or suction (13, 14).

Thulium lasers utilize thulium ions excited directly by high-power laser diodes. Two main types of thulium laser systems are available. Thulium:YAG lasers operate at a wavelength of 2010–2013 nm and are effective for both lithotripsy and soft tissue cutting and coagulation (12). In contrast, Thulium fiber lasers operate at a wavelength of 1940 nm and employ a fiber-based amplification system, enabling more precise and flexible energy delivery. This technology allows for higher pulse frequencies and lower pulse energies compared with thulium:YAG systems (11, 15).

These differences in design and emission characteristics result in distinct operational profiles, with clinical implications under investigation (11, 15). A potential limitation of both Holmium:YAG and thulium laser systems is the lack of integrated suction, important in the management of larger stone burdens.

The choice of energy sources in PCNL and mini-PCNL should take into account the nephroscope size, stone characteristics and surgeon experience. In this context, a comprehensive understanding of the available technologies becomes essential. Therefore, the aim of this article is to review the current literature on the various energy sources used for lithotripsy in PCNL and mini-PCNL, in order to assist urologists in selecting the most appropriate option for each clinical scenario.

## MATERIALS AND METHODS

A structured literature review was performed in PubMed and the Cochrane Library between June and September 2024. The search strategy included combinations of Medical Subject Headings (MeSH) terms and key words related to percutaneous nephrolithotomy and lithotripsy energy sources, including: "percutaneous nephrolithotomy", "ultrasonic lithotripsy", "pneumatic lithotripsy", "holmium laser", and "thulium laser", combined using the Boolean operators AND and OR.

Articles published between 2014 and 2024 were considered. A total of 95 records were initially identified. After initial review, 63 studies were excluded: 17 for involving pediatric populations, stones in anomalous or transplanted kidneys, and 46 for not providing direct comparative analysis, or use of energy sources other than ultrasonic, pneumatic, Holmium:YAG laser, or thulium laser.

A total of 32 articles supported this review, among these, 13 provided comparative data on different energy sources in PCNL and mini-PCNL and were included in the scope of this analysis (Figure-2).

**Figure 2:**
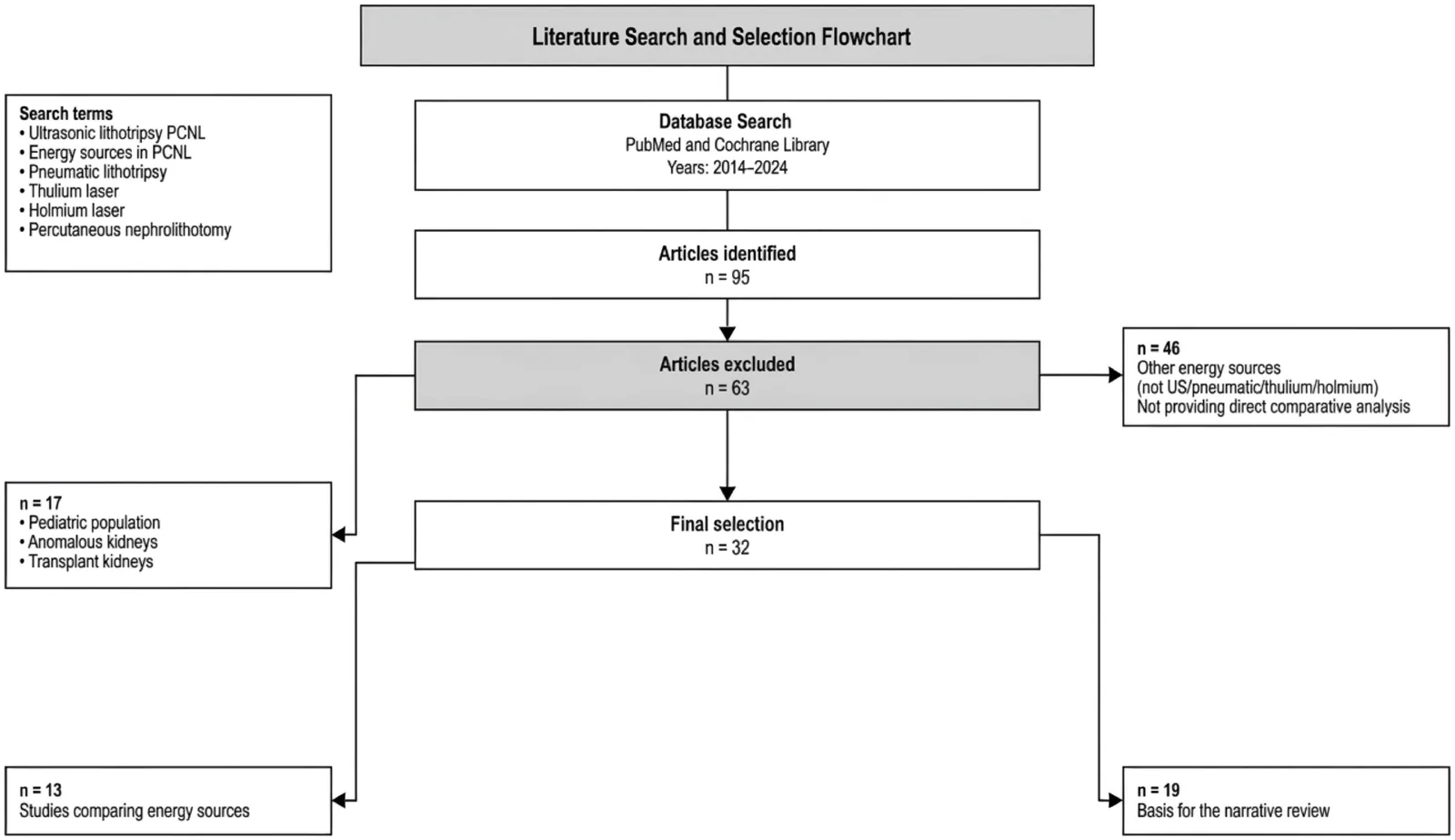
Literature Search and Selection Flowchart.

## RESULTS

This review comprised 32 studies, including 13 that performed comparative evaluations of different energy sources in PCNL and mini-PCNL, most of which were non-randomized. Comparative evidence comprised three randomized controlled trials (RCTs), a limited number of prospective non-randomized comparative studies, and predominantly retrospective studies.

In PCNL, two studies compared Holmium laser and ultrasonic lithotripsy, three evaluated ultrasonic versus pneumatic lithotripsy, and two assessed laser versus pneumatic energy sources. In the mini-PCNL subgroup, two studies compared laser and ultrasonic lithotripsy, one compared laser and pneumatic modalities, and three studies directly compared Thulium Fiber Laser (TFL) and Holmium:YAG laser.

## PCNL

The main outcomes evaluated were operative time, stone-free rate (SFR), blood loss, complications, and hospital stay.

Two studies (one retrospective and one randomized trial) compared Holmium:YAG laser and ultrasonic lithotripsy. Timm et al. (16) reported a shorter operative time with Holmium:YAG laser, whereas El-Nahas et al. (17) reported a shorter operative time in the ultrasonic group (130 ± 34 minutes vs. 148.7 ± 35 minutes). Stone-free rates, transfusion rates and complications were similar between groups. Blood loss was lower in the Holmium:YAG laser group (1.3 g/dL vs. 1.7 g/ dL). (Table-1).

**Table 1:** Comparative outcomes of Holmium:YAG laser and Ultrasonic lithotripsy in PCNL.

Study	Surgery Time (minutes), mean ± SD.	Stone-Free Rate (%)	Bleeding (hemoglobin deficit, g/ dL), mean ± SD or Estimated Blood Loss	Complications (% of patients)	Transfusion (%)	Median Hospital Stay (days)
Timm et al. (16)	Ho: 80.2 ± 16.7 US: 90.9 ± 28.1	Ho: 62.5 US: 55.5	NR	Ho: 9.1 G-II US: 6.25 G-IIIa	Ho: 9.1 US: 6.25	Ho: 3 US: 3
El-Nahas et al. (17)	Ho: 148.7 ± 35* US: 130.1 ± 34*	Ho: 66 US: 60	Ho: 1.3* US: 1.7*	Ho: 6 G-I, 11 G-II, 6 G-IIIa. US: 6 G-I, 17.9 G-II, 9 G-IIIa, 3 G-IVa	Ho: 11 US: 17	Ho: 5.1 US: 5.6
Radfar et al. (8)	US: 66.14 ± 30.11 Pneumatic: 68.17 ± 34.12	US: 90. Pneumatic: 93	US: 1.89 ± 0.47 Pneumatic: 1.76 ± 0.18	US: 11.95 G-I, 6.52 G-II. Pneumatic: 10.22 G-I, 7.95 G-II, 1.13 G-III.	NR	US: 3.19 ± 0.55 Pneumatic: 3.23 ± 0.77
Cauni et al. (18)	US: 43.5 Pneumatic: 77.13	US: 89.3 Pneumatic: 78	NR	US: 17.9 Pneumatic: 23	US: 0,071 Pneumatic: 0	NR
York et al. (19)	US: 152.2 Pneumatic: 129.8	US: 56.5 Pneumatic: 51.6	US: (>400mL) 4.4 Pneumatic: (>400mL) 6.7	US: 16.7 Pneumatic: 17.5	US: 2.9 Pneumatic: 4.0	US: 2.6 Pneumatic: 1.9

Ho: holmium laser; US: ultrasonic lithotripsy; SD: standard deviation; Hb: hemoglobin; min: minutes; NR: Not reported.

* statistically significant (p<0.05).

G-I, G-II, G-III, G-IIIa, G-IVa: Clavien-Dindo Grade I, Grade II, Grade III, Grade IIIa, and Grade IVa complications, respectively.

Three randomized studies (8, 18, 19) compared ultrasonic and pneumatic lithotripsy in PCNL. Operative time was shorter and stone-free rates were slightly higher in the ultrasonic group in two of the three studies; however, these differences did not reach statistical significance. Blood loss and complication rates were similar between groups. (Table-1).

Two studies (20, 21) compared Holmium:YAG laser and pneumatic lithotripsy in PCNL. Dua et al. (20) reported shorter operative time with pneumatic lithotripsy, whereas Hong et al. (21) did not confirm this finding. Stone-free rates were higher in the Holmium:YAG laser groups in both studies. Results regarding bleeding and hospital stay were inconsistent (Table-2).

**Table 2:** Comparative outcomes of Holmium laser and Pneumatic lithotripsy in PCNL.

Study	Surgery Time (minutes), mean ± SD.	Stone-Free Rate (%)	Bleeding (hemoglobin deficit, g/ dL), mean ± SD or patients with deficit > 0.5 g/dL	Complications (% of patients)	Transfusion (%)	Median Hospital Stay (days)
Dua et al. (20)	Ho: 59.7 ± 12.3* Pneumatic: 54.9 ± 13.2*	Ho: 94% Pneumatic: 88%	Ho: 13 patients Pneumatic: 11 patients	Ho: 14 Pneumatic: 16	Ho: 2 Pneumatic: 2	Ho: 5.9 ± 1.5 Pneumatic: 5.5 ± 1.7
Hong et al. (21)	Ho-YAG: 134.27 ± 53.38 Pneumatic: 137.7 ± 47.79	Ho-YAG: 59.09 Pneumatic: 48.15	Ho-YAG: 12 ± 15.42* Pneumatic: 19 ± 11.23*	NR	NR	Ho-YAG: 7.73 ± 2.75* Pneumatic: 8.94 ± 4.2*

Ho: Holmium laser; SD: standard deviation; Hb: hemoglobin; min: minutes; NR: Not reported.

* statistically significant (p<0.05).

## Mini-PCNL

Two studies (22, 23) compared ultrasonic lithotripsy and laser in mini-PCNL. Ultrasonic lithotripsy showed shorter operative time compared to Holmium:YAG laser, but longer operative time compared to TFL. Stone-free rates, blood loss, and complication rates were similar across groups. (Table-3).

**Table 3:** Comparative outcomes of Ultrasonic lithotripsy, TFL, and Holmium:YAG laser in mini-PCNL.

Study	Surgery Time (minutes), mean ± SD.	Stone-Free Rate (%)	Bleeding (hemoglobin deficit, g/ dL), mean ± SD	Complications (% of patients)	Transfusion (%)	Median Hospital Stay (days)
Akbulut et al. (22)	US: 107 ± 34.9 Ho-YAG: 113.5 ± 50.5	US: 68.1 Ho-YAG: 81.8	US: 1.15 ± 1.15 Ho-YAG: 0.93 ± 1.0	US: 18.2 G-IIIa Ho-YAG: 3.64 G-II, 9.1 G-III	NR	US: 79.6 ± 38.7 Ho-YAG: 74.2 ± 22.1
Patil et al. (23)	US: 32.48 ± 15.39 TFL: 28.63 ± 18.56	US: 96.6 TFL: 76.6	US: 1.19 ± 0.76 TFL: 0.99 ± 0.74	US: 10 G-II TFL: 6.67 G-II	US: 0. TFL: 0.	NR
Sharma et al. (24)	Ho-YAG: 52 ± 7.56 Pneumatic: 56 ± 7.84	Ho-YAG: 92.5 Pneumatic: 86.67	Ho-YAG: 0.88 ± 0.16 Pneumatic: 0.89 ± 0.23	NR	NR	Ho-YAG: 1.67 ± 0.45 Pneumatic: 1.73 ± 0.45

Ho-YAG: Holmium:YAG laser; US: ultrasonic lithotripsy; SD: standard deviation; Hb: hemoglobin; min: minutes; NR: Not reported; TFL: thulium fiber laser.

* statistically significant (p<0.05).

G-II, G-III, G-IIIa: Grade II, Grade III, and Grade IIIa Clavien-Dindo complications, respectively.

One study (24) compared Holmium:YAG laser and pneumatic lithotripsy in mini-PCNL, reporting comparable outcomes across all evaluated parameters (Table-3).

Three studies (25, 15, 26) compared Holmium:YAG laser and thulium fiber laser. TFL was associated with shorter operative time, lower blood loss, and reduced transfusion rates. (15, 25). Complication rates, hospital stay, and stone-free rates were similar between groups. In contrast, Vergamini et al. (26) reported shorter operative time with the Holmium:YAG laser (Table-4).

**Table 4:** Comparative outcomes of Holmium and Holmium:YAG Lasers versus Thulium Fiber Laser in mini PCNL.

Study	Surgery Time (minutes), mean ± SD.	Stone-Free Rate (%)	Bleeding (hemoglobin deficit, g/dL), mean ± SD.	Complications (% of patients)	Transfusion (%)	Median Hospital Stay (median-range, days)
Patil et al. (25)	Ho: 41.86 ± 13.2*. TFL: 23.68 ± 11.34*	Ho: 78.43 TFL: 68.63	Ho: 1.04 ± 0.445 TFL: 1.05 ± 0.711	Ho: 5.88 G-II TFL: 5.88 G-II	NR	NR
Mahajan et al. (15)	Ho-YAG: 68* TFL: 55*	Ho-YAG: 90.9 TFL: 94.9	Ho-YAG: 1.1* TFL: 0.9*	Ho-YAG: 6.1 G-I, 3 G-II TFL: 17.5 G-I, 1.8 G-II	Ho-YAG: 2.5* TFL: 0*	Ho-YAG: 1.5 TFL: 1.3
Vergamini et al. (26)	Ho-YAG: median of 67* TFL: median of 91*	Ho-YAG: 54.9 TFL: 67.3	Ho-YAG: 1.1 TFL: 1.0	Ho-YAG: 1.9 G-I, 1.9 G-II TFL 4 G-I, 2.2 G-II	NR	Ho-YAG: 1 (1-5) TFL 1 (1-3)

Ho-YAG: Holmium:YAG laser; SD: standard deviation; Hb: hemoglobin; min: minutes; NR: Not reported; TFL: thulium fiber laser.

* statistically significant (p<0.05).

G-I, G-II: Grade I and Grade II Clavien-Dindo complications, respectively.

Overall, most studies showed comparable stone-free rates and complication profiles across different energy sources, while operative time and bleeding outcomes varied between techniques.

## DISCUSSION

This narrative review highlights the advancements and challenges in energy sources for PCNL and mini-PCNL, based on the evaluation of 13 studies of direct comparative analysis, emphasizing their impact on procedural outcomes, patient safety, and clinical efficacy.

Ultrasonic lithotripsy is one of the most used energy sources in PCNL and has been the gold standard especially for large stones, with devices like the Lithoclast Master (or Lithoclast Ultra^®^), Trilogy^®^ and Shock Pulse-SE^®^ offering distinct characteristics that influence their advantages and disadvantages in renal stone treatment. The Lithoclast Master^®^, a device combining pneumatic and ultrasonic energy, with suction, is effective for fragmenting hard stones. In contrast, Trilogy^®^ represents a next-generation system that integrates ultrasonic and electromagnetic impact energy with active suction in a single probe. Studies have shown that Trilogy^®^ trends toward faster stone clearance rates due to its optimized energy delivery and suction capabilities, reducing the risk of fragment blockage when compared to the Lithoclast Master (7, 27, 28). It also offers customizable settings for different stone types, with low complications rates (7, 27, 28). However, Trilogy has reported drawbacks, including less ergonomic design, occasional probe failures and higher cost, potentially limiting its accessibility (28). The ShockPulse-SE, which combines continuous ultrasonic energy with intermittent ballistic impact and integrated suction, also indicates efficient performance in stone fragmentation and removal, with operative times and safety profiles comparable to or better than traditional devices (8, 29, 30). Experimental studies and animal models have shown that both Trilogy and ShockPulse-SE outperform the LithoClast Master in stone clearance efficiency, especially when larger-caliber probes are used (8, 30). In direct comparisons, Trilogy often achieves faster clearance times than ShockPulse-SE, although both are superior to previous-generation devices (8, 30). The choice between devices may depend on institutional factors, availability, cost, and surgeon preference, as both offer relevant technical advantages.

In this analysis, studies comparing Holmium:YAG laser and ultrasonic lithotripsy in standard PCNL (Table-1) demonstrate heterogeneous results. Operative time showed conflicting findings, with Timm et al. (16) reporting shorter procedures with the Holmium:YAG laser, whereas El-Nahas et al. (17) observed longer operative times with this modality. Stone-free rates were similar between groups. Regarding bleeding, El-Nahas et al. (17) reported a statistically significant lower hemoglobin drop in the Holmium:YAG group. Complications, transfusion rates and length of stay were comparable between modalities, with no clear advantage for either technique. Overall, these findings suggest equivalent efficacy, with a potential advantage of Holmium:YAG laser in reducing blood loss.

The comparison between ultrasonic and pneumatic lithotripsy in standard PCNL (Table-1), also revealed equivalent outcomes. Operative time was similar, although Cauni et al. (18) reported shorter procedures with ultrasonic devices, while York et al. (19) observed longer operative times compared to pneumatic lithotripsy. Stone-free rates were comparable across studies. Despite slight variations in reported hemoglobin drop, estimated blood loss, transfusion rates and overall complications were similar in both techniques. Overall, both modalities demonstrate similar efficacy and safety profiles, with no clear superiority.

Finally, studies comparing Holmium:YAG laser and pneumatic lithotripsy in standard PCNL (Table-2) suggest a trend toward improved stone clearance with laser technology. Operative time, complications and transfusion rates were similar between groups. Notably, Hong et al. (21) reported a statistically significant decrease in bleeding and hospital stay associated with Holmium:YAG lithotripsy. Overall, these findings suggest a potential advantage of Holmium:YAG laser in stone clearance.

In mini-PCNL, the use of ultrasonic energy is less prevalent compared to other modalities, primarily due to the limitations associated with probe size. Reduction in size of the ultrasound probes can lead to technical challenges, such as decreased power and efficiency in stone fragmentation, which are critical for effective lithotripsy (31). The study by Tabib et al. (32) highlights the performance of various lithotrites in a benchtop model, showing that mechanical lithotrites like the Trilogy 1.9 mm probe demonstrated superior stone clearance rates compared to smaller probes and laser fibers (32). These technical limitations can make ultrasound less favorable compared to other energy sources that can be more effectively miniaturized without significant loss of performance. Therefore, while ultrasound energy can be used in mini-PCNL, the preference for laser lithotripsy is often due to the superior performance and efficiency of these technologies. Operative time was similar between groups across studies. However, stone-free rates were inconsistent, with Akbulut et al. (22) favoring Holmium:YAG laser, whereas Patil et al. (23) reported higher clearance rates with ultrasonic lithotripsy. Hemoglobin drop tended to be slightly lower in laser groups but no significant differences in transfusion rates were observed, and complication rates were comparable. Hospital stay was also similar between modalities. As no statistical significance was obtained, these findings suggest that both techniques are effective, with no consistent superiority.

In the realm of laser lithotripsy for mini-PCNL, this analysis reveals a heterogeneous set of findings, where the Holmium:YAG laser is challenged by newer technologies, particularly the Thulium Fiber Laser.

The Holmium:YAG laser is a cornerstone in mini-percutaneous nephrolithotomy due to its proven efficacy and versatility. These systems operate at a wavelength of 2120 nm, providing efficient stone fragmentation and tissue ablation with minimal thermal damage. High-power lasers offer adjustable pulse durations and high-power settings where low power lasers, on the other hand, are known for their reliability and low cost, making it a popular choice for urologists performing both conventional and miniaturized PCNL. However, the results from comparative studies suggest that its dominance is not absolute. While equivalent stone-free rates (SFR) and complications between Holmium and ultrasonic lithotripsy are reported, the comparison with TFL is more informative. Two studies demonstrated a statistically significant reduction in operative time with TFL, and one of them observed a statistically significant decrease in bleeding and transfusion rates (15, 25). This may be explained by the TFL's higher absorption in water, leading to potentially less thermal spread to surrounding tissue.

Conversely, the study by Vergamini et al. (26) presented a contrasting outcome, reporting a shorter surgical time for a Holmium:YAG laser system with MOSES technology compared to TFL. This finding suggests an important aspect: the performance of a laser is not solely defined by its wavelength but also by its specific pulse modulation technology (e.g., MOSES, Vapor Tunnel) and the surgeon's technique. This conflict in the evidence indicates that the choice between Holmium systems with pulse modulation and TFL may, in some cases, be influenced by factors beyond the laser source itself.

Thulium Fiber Laser has gained traction due to its low weight and efficient profile. Operating at a wavelength of 1940 nm, TFL systems excel in water absorption, enabling finer stone dusting and reduced retropulsion during percutaneous and mini-percutaneous nephrolithotomy. On the other hand, TFL has a lower peak-power, making it less efficient in hard stones. The available evidence suggests TFL's favorable profile for mini-PCNL. It's better in operative time and bleeding align with its theoretical advantages. The more efficient "dusting" capability of TFL appears to be well suited for procedures utilizing suction sheaths, where the goal is to clear the field continuously without the need for fragment removal. However, it is crucial to balance this enthusiasm with practical considerations. The TFL's lower peak power, compared to Holmium:YAG, may translate into less efficiency when facing very hard stones. This potential limitation was not directly assessed in the included studies but remains a relevant factor for surgical planning.

As for the Thulium:YAG laser, the current body of evidence is insufficient to draw definitive conclusions about its role in PCNL and mini-PCNL. This review identified a notable lack of clinical studies directly comparing Tm:YAG with Ho:YAG or TFL in the percutaneous setting. While its wavelength of 2013 nm and uniform pulse profile present a relevant theoretical profile, the absence of robust clinical data prevents any meaningful comparison of its efficacy and safety against the established options. Therefore, its promise, though attractive, remains largely unvalidated in clinical practice for this specific application.

In summary, while the Holmium:YAG laser continues to be a reliable and effective standard, the TFL is associated with improved outcomes in some parameters for mini-PCNL. The choice between them may depend on stone characteristics, available technology, and surgeon expertise. The role of Tm:YAG, however, awaits further clinical validation.

Despite these advancements, several limitations in the current evidence must be acknowledged. The heterogeneity of study designs, patient populations, and surgical settings introduces variability that may limit the generalizability of findings. Future research should prioritize large-scale randomized controlled trials comparing newer laser technologies to the traditional methods like ultrasound and ballistic lithotrites. Cost-effectiveness analyses and studies incorporating patient-reported outcomes will also be essential to guide clinical decision-making.

## CONCLUSIONS

This narrative review elucidates the comparative efficacy, safety, and practical considerations of various energy sources in PCNL and mini-PCNL, providing evidence-based insights to guide clinical decision-making.

In PCNL, ultrasonic lithotripsy and Holmium-Yag laser demonstrates comparable SFR with reduced blood loss with Holmium laser. Ultrasound provides shorter operative times and higher SFR when compared to pneumatic devices. Holmium laser also demonstrated superior stone-free rates compared to pneumatic lithotripsy.

In mini-PCNL ultrasound and Laser are equivalent with similar SFR, surgery times and complications. TFL provides reduced operative time, bleeding, and transfusion rates compared to Holmium:YAG, not evidenced when Holmium:YAG is coupled with MOSES technology.

## Data Availability

All data generated or analysed during this study are included in this published article

## ABBREVIATIONS

PCNL: Percutaneous nephrolithotomy
mini-PCNL: Mini-percutaneous nephrolithotomy
Ho:YAG / Ho-YAG: Holmium:yttrium-aluminum-garnet (laser)
TFL: Thulium fiber laser
Tm:YAG: Thulium:yttrium-aluminum-garnet (laser)
SFR: Stone-free rate
RCT: Randomized controlled trial
NHANES: National Health and Nutrition Examination Survey
MOSES: Modulated Output Synchronized Energy System (pulse modulation technology for Holmium laser)
EMS: Electro Medical Systems (LithoClast manufacturer)
NR: Not reported
SD: Standard deviation
G-I, G-II, G-IIIa, G-IVa: Clavien-Dindo complication grades I, II, IIIa, and IVa
YAG: Yttrium-aluminum-garnet
